# Application of vascularized fibular graft for reconstruction and stabilization of multilevel cervical tuberculosis: A case report

**DOI:** 10.1097/MD.0000000000009382

**Published:** 2018-01-19

**Authors:** Jian Zhang, Wen-Si He, Cheng Wang, Yi-Guo Yan, Zhi-Hua Ouyang, Jing-bo Xue, Xue-Lin Li, Wen-Jun Wang

**Affiliations:** a Department of Hand and Micro-surgery; b Department of Spine Surgery, the First Affiliated Hospital, University of South China, Hengyang, Hunan, China.

**Keywords:** cervical tuberculosis treatment, free fibular flap, long-term follow-up, reconstructive surgery, review of grafts types

## Abstract

Multilevel cervical reconstruction and fusion after cervical tuberculosis has always been a challenge. The current implantation materials for cervical fusion, including titanium mesh, cage, and plate are limited by its inferior biological mechanical characteristics and the properties of the metallic material. This has led to the increased risk of recurrent infection after surgery. In addition, the unique nature of tuberculosis infection results in the low rate of cervical fusion and high risk of recurrence. This case report presents 1 patient who suffered from long segmental cervical tuberculosis and had reconstruction surgery using a vascularized fibula graft. The patient had successful graft incorporation 3 months postsurgery and was followed-up for 30 months. In this review, we detail the advantages of using vascularized fibular grafts and compare it with other types of grafts.

## 1 Introduction

There are various reasons to perform multilevel cervical fusion, including the failure of posterior arthrodesis, tuberculosis (TB) infections, severe spinal deformity, and total or partial resection of spinal tumors. The traditional anterior approach to cervical fusion has limited its application in multilevel cervical fusion. Taylor et al^[1]^ first reported in 1975, the vascularized fibular graft (VFG) that is widely utilized today in the reconstruction of limb bone defects. Hara et al^[2]^ reported a case that had a successful outcome using VFG in midfoot reconstruction. It was also reported that VFG for posttrauma bone defects was efficacious.^[3,4]^ In addition, numerous reports have demonstrated the advantage of VFG after resection of bone tumor or necrotic tissue.^[5–8]^ In upper extremities, the VFG also have certain value.^[9,10]^ However, in cervical reconstruction, a large proportion of case reports that have been published involve spinal orthopedic and tumor resections.^[11–14]^ It is extremely rare to find published reports that used VFG for long segmental cervical reconstruction in severe cervical TB. Our study review presents a successful case of VFG for cervical reconstruction in a patient with spinal tuberculosis.

## 2 Conclusion

The VFG is an excellent choice for reconstruction of multilevel cervical defects due to spinal tuberculosis. The rapid and ideal fusion, as well as the lower risk of tuberculosis recurrence in this case study indicates that VFG may be a favorable surgical option for long segmental cervical tuberculosis.

## 3 Case report

### 3.1 Ethics statement

The patient agreed to the study and signed the informed written consent form. The study was approved by the Research Ethics Committee of the First Affiliated Hospital, University of South China.

### 3.2 Medical history

A 45-year-old woman suffered from occipital and back, neck pain, which was worse on the left side for about 11 months. The symptoms were accompanied with low-grade fever and repeated night sweats. The patient underwent conservative treatment in a local hospital but the symptoms persisted and began to further deteriorate for a month before being referred to our hospital. In addition, she showed signs of dysphagia. During the course of the disease she had loss of appetite and her weight significantly decreased by approximately 15 kg. The patient had no chest band tightness but felt like she was “walking on cotton.” Her trunk and limb movements were normal.

### 3.3 Physical examination

Her cervical curvature was straight and her cervical muscles were strained. Regarding neurological examinations, she had a slight enhancement in biceps, triceps, and radial reflex. All other physical examinations were normal.

### 3.4 Laboratory examinations

The patient's liver, kidney, coagulation function, and blood electrolytes were in the normal range. Erythrocyte sedimentation rate (ESR) was 67 mm/h and hemoglobin (Hb) was 107 g/L which suggested mild anemia.

### 3.5 Radiographic assessment

**Figure 1:**
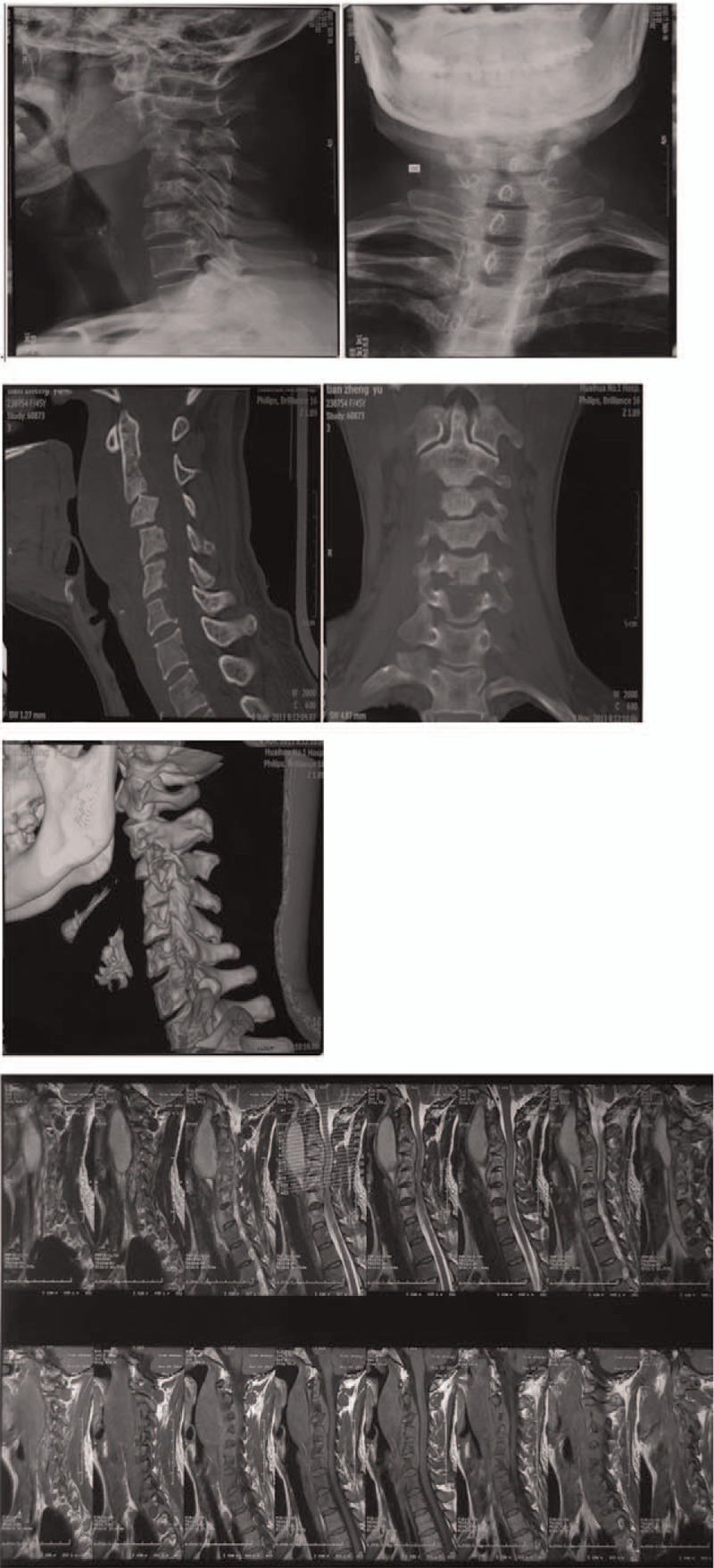
The preoperative scans reveal reverse spondylolisthesis of the C3 vertebral body and destruction of the C5, C6, and C7 vertebral body (VB) and presence of paravertebral abscess.

C-spine x-ray is presented in Fig. 1 and exhibited a reverse slip of C3 vertebral body. Computed tomography (CT) and magnetic resonance imaging (MRI) showed destruction of C5, C6, C7 vertebral body and formation of paravertebral abscess. The following diagnosis was made; multilevel cervical TB and abscess presence; reverse slip of C3 vertebral body; bone destruction of C5, C6, and C7 vertebral body.

### 3.6 Preparation for surgery

The patient was treated with anti-TB drug therapy (isoniazide, 300 mg, Qd, intravenous drip; rifampicin, 0.45 g, taken orally, Qd; pyrazinamide, 0.75 g, taken orally, Qd; ethambutol, 0.75 g, taken orally, Qd) for 1 week with ESR follow-up and the relevant laboratory examinations, until these indicators were in the normal range.

### 3.7 Surgery

**Figure 2:**
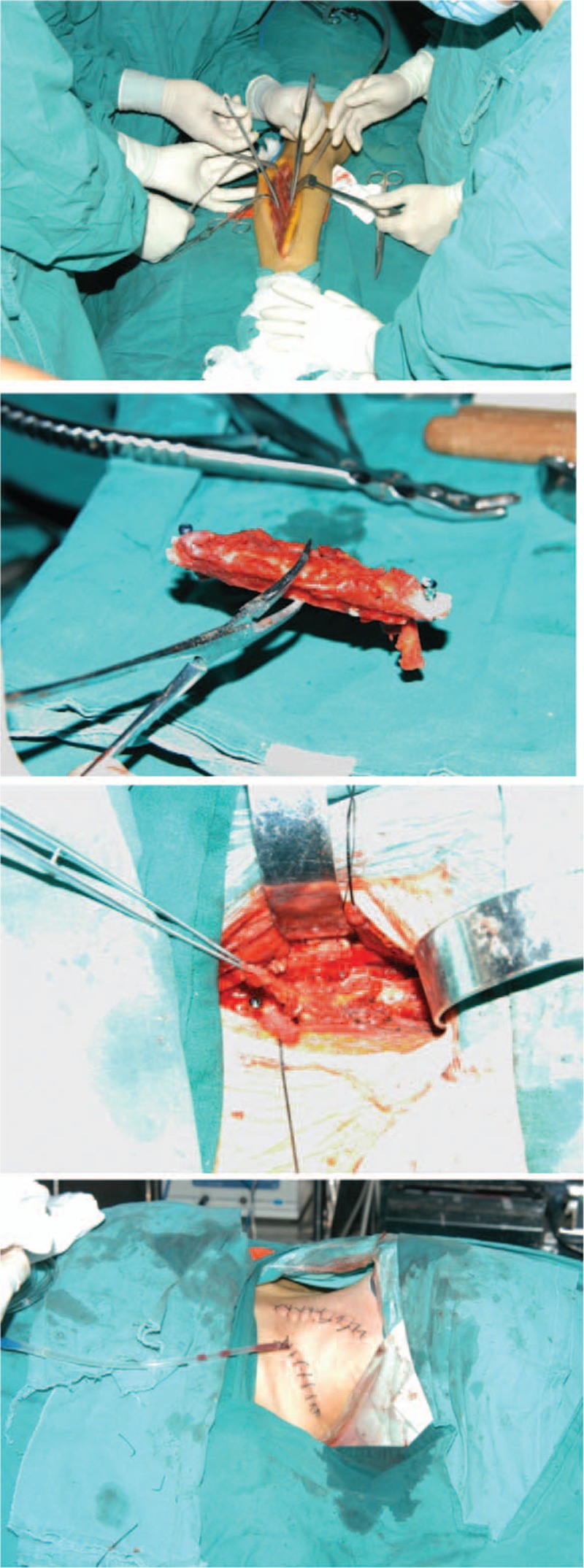
The intraoperative scans demonstrates the removal of the intact vascularized fibular.

The surgery was performed in 2 stages. Intraoperative images are presented in Fig. 2.

#### 3.7.1 Stage 1 of the surgery

The first stage of the surgery was the posterior cervical internal fixation and the length interception of the vascularized fibular. Patient was immobilized using the cephalostat in the prone position. The posterior region of the neck and left leg was disinfected and skin incision was performed at the level of the posterior midline. The muscles, vessels, and fascia were dissected to expose the lateral mass of the C2 to C7 vertebral body. C2 and C7 were immobilized using 2 pairs of pedicle screws and C4 and C6 VB were immobilized using lateral mass screws. A titanium rod was inserted based on the physiological curvature and inserted into the groove with screws and fixed with pedicle screw caps and subsequently the surgery wound was closed.

#### 3.7.2 Stage 2 of the surgery

Ten days postoperation, stage 2 of the surgery was performed. This stage was divided into 2 steps. The first step was the removal of the fibular. The fibular was harvested for the appropriate length and vascular pedicle under the guidance of an air pressure tourniquet. An incision about 15 cm was made along the posterior margin of the fibular and then the intramuscular space of the soleus and peroneus longus was exposed by dissecting skin and muscles carefully. Using the intramuscular space as point of entry, the peroneal artery and vein was located at the back of the tibialis posterior and inner side of the flexor hallucis longus and carefully protected. The peroneus longus and brevis were dissected carefully while preserving the straticulate sleeve on the fibula. Approximately, 9.5 cm of the fibula was excised by osteotomies. The middle and distal part of the fibula was selected as the graft because the diameter of this section was small and uniform as well as for the preserving of the vascular pedicle. A uniform, thin fibular graft is advantageous because it decreases the risk of tracheal and esophagus compression. After step 1, the patient was placed in a supine position and step 2 of the surgery was performed. Preoperative disinfection was performed in the anterior region of the neck. Incision was made at the left side of the anterior cervical and avoided the trachea and carotid artery. Precautions were taken to protect the trachea and esophagus during the dissection of the sternocleidomastoid and omohyoid. The mass of destruction of all intervertebral discs and vertebras were exposed. The C3 vertebra had spondylolysis combined with the failure and instability of C4, C5, and C6. The fluctuation would be felt after drawing back the lower jaw using a drag hook. The drained was about 25 mL. After performing these steps, we performed corpectomis and then the C3, C4, C5, and C6 vertebras and adjoining intervertebral discs (IVDs) were decompressed and debrided. During this process, most of the posterior longitudinal ligaments were dissected and the endorhachis was exposed for complete decompression. The surgical area was irrigated using hydrogen peroxide and normal saline combined with anti-tuberculosis drugs. The vascularized fibula was grooved into a trapezoid shape in the C2 and C7 vertebral bodies and then the fibular graft was shaped based on the defect of the recipient area. The fibular graft was inserted tightly in front of the cervical. The graft was fixed using 17 mm screws on the upper and lower surface. After surgery, C-shaped x-ray was performed and showed that the fixation and embedding of the graft was satisfactory. Next, we performed vessel anatomosis. The transverse cervical artery and external jugular vein were located at the superior border of the omohyhoid. Full dissociation must be performed carefully to retain the integrity of vessel pedicle. The free artery and vein were excised and ligated at the distal end. Anatomosis was performed between the transverse cervical artery and peroneal artery together with the peroneal vein and external jugular vein. The vessels were checked to be unobstructed by recanalization. There was no vessel thrombosis and vasospasm after 30 minutes of observation. The retropharyngeal and throat were filled with gelatin sponges that released isoniazid and streptomycin. A drainage tube was placed and the wound was closed.

The duration of the surgery was 6 hours. After the surgery, blood pressure at the supradavicular area of the anterior cervical returned to normal.

### 3.8 Postoperative treatment

**Figure 3:**
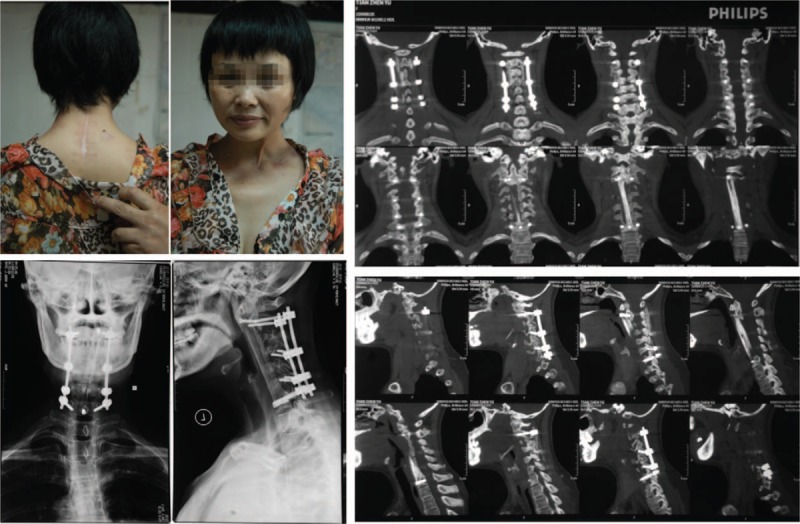
Scans 6 months postsurgery demonstrates strong internal fixation in the correct position and good fusion.

**Figure 4:**
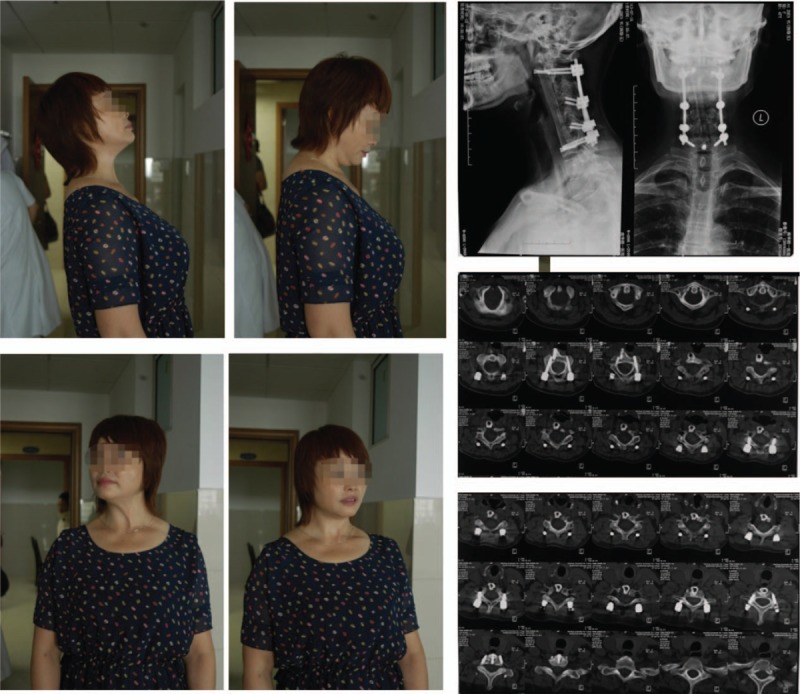
Scans at 18 months postsurgery showed satisfactory recovery of cervical function. Patient performed anteflexion, rear protraction, and lateral neck bending. X-ray and CT scans demonstrated no recurrence of TB and the internal fixation and fusion were excellent. CT = computed tomography, TB = tuberculosis.

**Figure 5:**
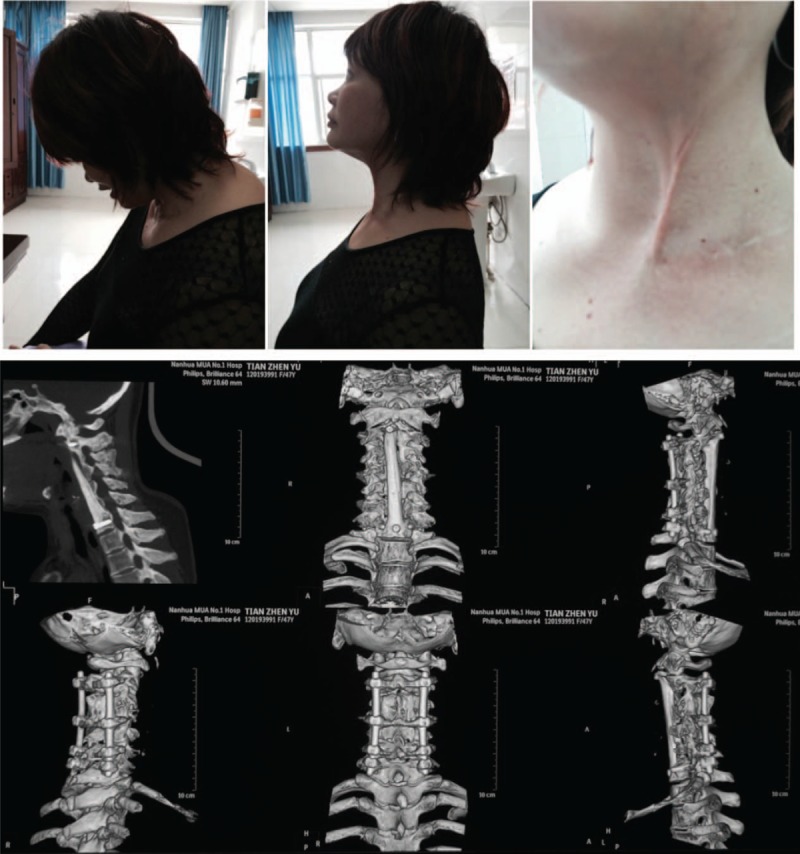
The radiographic image analysis at 28 months post-op shows strong internal fixation and good fusion. There were no signs of TB recurrence. The location of the internal fixation device was proper. Postoperative outcomes were measured using the patients’ cervical range of motion (ROM). TB = tuberculosis.

After the surgeries were completed, the patient was treated using anti-TB drugs for 2 weeks as well as liver-protecting therapy simultaneously. The dose of anti-TB drugs were the same as that administered pre-op. Postoperatively, the patient recovered from low-grade fever and symptoms of occipital and back-neck pain improved. X ray and CT scans postsurgery revealed that the location of the fixation system and fibular graft were not displaced or extruded. The patient began functional exercises out of bed with the assistant of a cervical orthosis by day 10 postsurgery and was discharged after 27 days. After 3 months, the patient was followed-up with CT scans and x-rays. The results showed good fibular graft incorporation. At 6, 18, and 28 months’ of follow-up, CT scans and x-rays (Figs. 3–5) showed complete incorporation of the fibular grafts. In addition, the patient had a good range of motion and functionally recovered after surgery. The patient was able to perform moderate lateral bending, anteflexion as well as rear protraction with no signs of abnormal sensation or pain in the upper limbs. Radiography showed excellent cervical fusion. In addition, after each follow-up, there were no signs of complications or pain in the donor site, as well as any recurrence of infection.

## 4 Discussion

Spinal TB is the most common bone and joint tuberculosis and accounts for approximately 50 percentage of all osteoarticular TB.^[15]^ Patients who suffer from spinal TB are usually malnourished or the frail elderly with systemic diseases. Spinal TB may induce the destruction of vertebral bodies and result in spinal deformity. In more severe cases, the spinal canal and nerve root are involved leading to spinal cord compression and the corresponding clinical symptoms.^[16]^ Traditional, conservative chemotherapy is used to treat patients who are at the early stages of the disease process and have no severe complications such as kyphosis or neurological deficit. However, surgical intervention is usually required for patients that have severe kyphosis, pan vertebral lesions, neurological deficits, or therapeutically refractory disease.^[17]^ Radical debridement of destructed tissues and bones has been demonstrated to be efficacious in preventing further deterioration, including recurrence, spread of infections, and late-onset paraplegia. However, radical debridement has limitation for treating long segmental cervical TB, as it necessitates the reconstructing the stability and biomechanical features of the cervical. Reconstruction of the spinal instability is crucial to increase the quality of life after surgical intervention. Traditional titanium plates combined with titanium meshes that are filled with allograft bone is only suitable for short segmental fusion. It is difficult to find long titanium meshes and plates for long segmental cervical reconstruction. Thus, it is vital to find new methods for reconstruction of long segmental cervical defects. Taylor et al. first demonstrated in 1975,^[1]^ that VFG is a new effective method for reconstruction surgery and exhibits remarkable biologic, mechanical, and rapid fusion properties. In this study, our patient underwent this method for the reconstruction of long segmental cervical tuberculosis.

It has been reported that autogenous bone grafts are superior for spinal fusion compared with other types of bone grafts. Cacellous bone grafts are used to repair small bony defects, whereas corticocacellous bone grafts are required to provide biomechanical functions.^[13]^ Inflammation and infections always leads to the fusion failure in case of cacellous bone grafts.^[18]^

At present, bone grafts used for reconstruction of the spine are usually from the rib, iliac, or fibula. The application of these type of grafts depend on the size, location, type of defect that needs to be repaired as well as the mechanical recovery that is required.^[13]^ The use of rib grafts are limited because of its oval shape in cross-section, curved, and relatively thin cortex.^[19–21]^ In addition, the rib is too weak to provide sufficient biomechanical superiority. Iliac is another popular graft source for spinal reconstruction. However, there are limitations in harvesting iliac and may result in persistent pain in the donor site.^[22]^ Similarly, iliac grafts have its drawbacks for its inadequate inherent strength to resist fracture and segmental collapse.^[11]^ In addition, the length of the iliac that is harvested is too short to meet the requirements for this operation. In our patient, the graft needed for multilevel cervical reconstruction needed to be long and the length may be variable depending on the surgery required. These requirements limited the application of the iliac and rib as bone grafts. Fibular is an ideal graft and is advantageous for the following reasons; can be easily harvested and, the surrounding cortex is strong to resist fracture and collapse. The grafts from the fibular can be as long as 20 cm while available iliac and rib cannot reach this length. Wittenberg et al^[23]^ had compared the strength of various types of non-vascularized grafts and found that the fibular strut grafts were superior for strength. In our patient, grafts were harvested from the middle and lower shafts of the fibular. This is the part of the fibular that is uniform, small in diameter and size, and can reduce the risk of extrude from the fibular graft strut. In some cases after surgery, the fibular strut may displace and result in compression to the esophagus and trachea anteriorly. Correctly choosing the fibular section that is harvested can effectively avoid this problem.

There are many differences between vascularized and non-vascularized bone grafts. In many cases, the rate of fusion is higher in patients with vascularized bone grafts. Vascularized bone grafts has approximately 80% to 90% fusion rate and is higher than non-vascularized bone grafts.^[24,25]^ The fusion pattern of non-vascularized bone grafts is “creeping substitution” and requires at least 2 years to achieve complete fusion.^[26,27]^ Six months post-op is the crucial period for non-vascularized bone graft to fuse.^[26]^ In canine studies, vascularized grafts were superior than non-vascularized grafts when compared with increased stiffness and stability.^[28,29]^ Studies have indicated that vascularized grafts contain more viable osteocytes and hence avoid massive bone remodeling thus reducing the risk of stress fractures. In addition, hypertrophy may occur in vascularized bone grafts in response to stress while it is absent in non-vascularized bone grafts.^[30]^ Intact endosteal and periosteal vascular blood flow is the main reason for maintaining cellular viability in bone grafts.^[31]^ Importantly, vascularized bone grafts exhibit better reconstructive features compare to non-vascularized bone grafts even under infections.^[30]^ In our patient, scans as well as the observations during surgery showed extensive destruction of bone and massive pus-like substance. Not surprisingly, the patient complained of having repeated low-grade fever. These symptoms suggested the possibility of tuberculosis infection. Non-vascularized bone grafts could not have been used in our patient as it shows poor resistance to infections and may lead to fusion failure.

The choice of blood vessels was another challenge in this patient. In the majority of previous cases, the superior thyroid artery was selected for anterior cervical fusion. This is because of the ideal location and quality of the recipients’ vessels in the cervical area have made revascularization easier during surgery.^[31]^ However, in our patient, due to tuberculosis infection, the soft tissue bed was compromised and conventional recipient vessels were too fragile to provide favorable blood flow. The blood flow of the transverse cervical artery was strong than other arteries and was selected. The selection criteria were to avoid selecting fragile arteries which may result in the failure of revascularization and increase the risk of recurrent infection of tuberculosis. The vein in cervical area that is antosomed to peroneal vein is external jugular vein which is similar as Winter et al's research.^[31]^

Previous studies have demonstrated that infection is the main factor for non-fusion or delayed fusion in bone reconstruction.^[32]^ The biggest challenge that may result in non-fusion in our patient was TB infection. To eradicate TB infection, specific drug therapy is required. The location of infection in the cervical area has limited the effect of these specific anti-tuberculosis drugs. This presented a tough scenario for bone fusion in our patient. Numerous studies have demonstrated that VFGs have a significant ability to resist infection and achieve stable and rapid fusion. The results of our patient follow-up showed no signs of TB recurrence and the fusion was excellent. Our case study further demonstrates that fibular graft's ability to resist infections.

Another advantage of our study compared with previous studies is the use of posterior internal fixation. The posterior internal fixation provided stronger mechanical support until the bony fusion was complete. This extra mechanical support shared the mechanical stress that is imposed to the fibular strut graft, thus decreasing the risk of stress fracture. In addition, the reverse slip of C3 vertebral body can be corrected by using posterior internal fixation.

Some limitations and challenges are present in the application of fibular grafts. One of the issues is donor site mobility. The location of the fibular that is resected too distally may result in functional valgus of the ankle joints.^[33]^ Gaskill et al^[34]^ studied 946 VFGs cases between 1990 and 2006. The study demonstrated that the major complications after surgery were rare and the minor complications were controllable. More rigorous clinical studies are still required for VFG to be widely used in surgery.

## 5 Conclusion

The VFG is an excellent choice for reconstruction of multilevel cervical defects due to spinal tuberculosis. The rapid and ideal fusion as well as the lower risk of tuberculosis recurrence in this case indicates that this method may offer a new treatment strategy for long segmental cervical tuberculosis.
